# Supplementary material for: Land cover and space use influence coyote carnivory: evidence from stable-isotope analysis
Source: PeerJ. 2024 Jun 4;12:e17457. doi: 10.7717/peerj.17457 (PMC11160434; doi:10.7717/peerj.17457)
Supplement: Supplemental Information 2 — Some fruit, small mammal, and white-tailed deer data were collected from our study area but most data were taken from nearby studies. [file peerj-12-17457-s002.docx]

Table S2: Prey isotope values used to estimate diet fractions for coyotes via stable-isotope mixing models. Some fruit, small mammal, and white-tailed deer data were collected from our study area but most data were taken from nearby studies.

| Food source | Location | Data type | δ^13^C | δ^15^N | Source |
| --- | --- | --- | --- | --- | --- |
| Blackberry01 | Georgia | Individual | -25.2 | 0.7 | This study |
| Blackberry02 | Georgia | Individual | -27.9 | 1.1 | This study |
| Blackberry03 | Georgia | Individual | -26.5 | 1.4 | This study |
| Blackberry04 | Georgia | Individual | -26.3 | 0.5 | This study |
| Blackberry05 | Georgia | Individual | -28.1 | 0.9 | This study |
| Blackberry06 | Georgia | Individual | -29.2 | 0.0 | This study |
| Persimmon01 | Georgia | Individual | -29.0 | 0.0 | This study |
| Persimmon02 | Georgia | Individual | -26.3 | 1.2 | This study |
| Persimmon03 | Georgia | Individual | -28.9 | 0.0 | This study |
| Persimmon04 | Georgia | Individual | -30.2 | 0.0 | This study |
| Persimmon05 | South Carolina | Individual | -28.3 | -2.8 | Carter et al., 2021 |
| Persimmon06 | South Carolina | Individual | -27.6 | -2.3 | Carter et al., 2021 |
| Pokeberry01 | South Carolina | Individual | -29.5 | 3.7 | Carter et al., 2021 |
| Pokeberry02 | South Carolina | Individual | -26.1 | -2.9 | Carter et al., 2021 |
| Muscadine01 | South Carolina | Individual | -26.4 | -7.0 | Carter et al., 2021 |
| Muscadine02 | South Carolina | Individual | -28.2 | -5.8 | Carter et al., 2021 |
| Muscadine03 | South Carolina | Individual | -25.4 | -4.9 | Carter et al., 2021 |
| Muscadine04 | South Carolina | Individual | -25.0 | -8.9 | Carter et al., 2021 |
|  |  | **Fruit study mean** | **-27.5** | **-1.4** |  |
|  |  | **Fruit study SD** | **1.6** | **3.4** |  |
|  |  |  |  |  |  |
| Grasshopper01 | Georgia | Shrub mean | -27.7 | 1.7 | Brittain, 2009 |
| Grasshopper02 | Georgia | Shrub mean | -26.0 | 0.4 | Brittain, 2009 |
| Grasshopper03 | Georgia | Oak forest mean | -26.0 | 0.5 | Brittain, 2009 |
| Grasshopper04 | Georgia | Pine forest mean | -27.0 | 0.8 | Brittain, 2009 |
| Grasshopper05 | Georgia | Shrub mean | -21.8 | 2.4 | Brittain, 2009 |
| Grasshopper06 | Georgia | Oak forest mean | -14.5 | 0.1 | Brittain, 2009 |
|  |  | **Insect study mean** | **-23.1** | **0.9** |  |
|  |  | **Insect study SD** | **5.0** | **0.9** |  |
|  |  |  |  |  |  |
| Hispid cotton rat01 | Georgia | Individual | -25.3 | 3.6 | This study |
| Hispid cotton rat02 | Georgia | Individual | -25.3 | 3.5 | This study |
| Cotton mouse01 | Georgia | May milo mean | -22.5 | 3.1 | Rush et al., 2014 |
| Cotton mouse02 | Georgia | May non-milo mean | -24.5 | 3.9 | Rush et al., 2014 |
| Cotton mouse03 | Florida | May milo mean | -18.7 | 5.3 | Rush et al., 2014 |
| Cotton mouse04 | Florida | May non-milo mean | -22.8 | 4.7 | Rush et al., 2014 |
| Least shrew01 | Georgia | May milo mean | -22.4 | 6.3 | Rush et al., 2014 |
| Least shrew02 | Florida | May milo mean | -23.5 | 5.6 | Rush et al., 2014 |
| Least shrew03 | Florida | May non-milo individual | -23.7 | 4.4 | Rush et al., 2014 |
| Hispid cotton rat03 | Georgia | May milo mean | -19.0 | 2.9 | Rush et al., 2014 |
| Hispid cotton rat04 | Georgia | May non-milo mean | -23.8 | 2.9 | Rush et al., 2014 |
| Hispid cotton rat05 | Florida | May milo mean | -17.1 | 4.3 | Rush et al., 2014 |
| Hispid cotton rat06 | Florida | May non-milo mean | -24.0 | 3.5 | Rush et al., 2014 |
| Eastern woodrat01 | Georgia | May milo treated individual | -26.0 | 1.6 | Rush et al., 2014 |
| Eastern woodrat02 | Florida | May milo treated individual | -26.3 | 2.6 | Rush et al., 2014 |
| Eastern woodrat03 | Florida | May non-milo treated individual | -25.2 | 4.1 | Rush et al., 2014 |
| Eastern cottontail | Texas | Individual | -17.5 | 4.0 | Smith et al., 2014 |
| Eastern cottontail | Texas | Individual | -20.7 | 2.3 | Smith et al., 2014 |
| Eastern cottontail | Texas | Individual | -17.6 | 8.3 | Smith et al., 2014 |
| Eastern cottontail | Texas | Individual | -18.4 | 2.7 | Smith et al., 2014 |
| Eastern cottontail | Texas | Individual | -24.3 | 2.0 | Smith et al., 2014 |
| Eastern cottontail | Texas | Individual | -21.2 | 2.6 | Smith et al., 2014 |
| Eastern cottontail | Texas | Individual | -23.4 | 2.5 | Smith et al., 2014 |
| Eastern cottontail | Texas | Individual | -20.4 | 5.7 | Smith et al., 2014 |
| Eastern cottontail | Texas | Individual | -18.0 | 4.9 | Smith et al., 2014 |
| Eastern cottontail | Texas | Individual | -21.5 | 3.1 | Smith et al., 2014 |
| Eastern cottontail | Texas | Individual | -21.8 | 3.2 | Smith et al., 2014 |
| Eastern cottontail | Texas | Individual | -20.8 | 3.6 | Smith et al., 2014 |
| Eastern cottontail | Texas | Individual | -20.9 | 3.1 | Smith et al., 2014 |
| Eastern cottontail | Texas | Individual | -21.9 | 2.8 | Smith et al., 2014 |
| Eastern cottontail | Texas | Individual | -20.9 | 3.0 | Smith et al., 2014 |
| Eastern cottontail | Texas | Individual | -17.8 | 5.1 | Smith et al., 2014 |
|  |  | **Small mammal study mean** | **-21.8** | **3.8** |  |
|  |  | **Small mammal study SD** | **2.7** | **1.4** |  |
|  |  |  |  |  |  |
| Northern bobwhite01 | Georgia | May milo treated mean | -18.1 | 4.8 | Rush et al., 2014 |
| Northern bobwhite02 | Georgia | May non-milo treated mean | -26.6 | 4.4 | Rush et al., 2014 |
| Northern bobwhite03 | Florida | May milo treated mean | -19.0 | 5.8 | Rush et al., 2014 |
| Northern bobwhite04 | Florida | May non-milo treated mean | -24.1 | 4.5 | Rush et al., 2014 |
| Eastern wild turkey01 | Florida | Individual | -19.2 | 5.7 | Hutchinson et al., 2016 |
|  |  | **Game bird study mean** | **-21.4** | **5.0** |  |
|  |  | **Game bird study SD** | **3.7** | **0.7** |  |
|  |  |  |  |  |  |
| White-tailed deer01 | Georgia | Individual | -24.4 | 3.0 | This study |
| White-tailed deer02 | Georgia | Individual | -24.1 | 3.9 | This study |
| White-tailed deer03 | Georgia | Individual | -25.3 | 3.4 | This study |
| White-tailed deer04 | Georgia | Individual | -23.9 | 2.6 | This study |
| White-tailed deer05 | Alabama | Individual | -22.2 | 4.5 | Cormie & Schwarcz, 1994 |
| White-tailed deer06 | Mississippi | Individual | -23.1 | 2.7 | Cormie & Schwarcz, 1994 |
| White-tailed deer07 | Louisiana | Individual | -22.0 | 5.7 | Cormie & Schwarcz, 1994 |
|  |  | **Deer study mean** | **-23.6** | **3.7** |  |
|  |  | **Deer study SD** | **1.2** | **1.1** |  |
|  |  |  |  |  |  |
| Corn feed01 | Texas | Mean | -11.6 | 2.6 | Darr & Hewitt, 2008 |
| Corn feed02 | Texas | Mean | -11.7 | 2.2 | Darr & Hewitt, 2008 |
| Corn feed03 | New Mexico | Mean | -11.9 | 4.6 | Boggie et al., 2018 |
|  |  | **Corn feed study mean** | **-11.7** | **3.1** |  |
|  |  | **Corn feed study SD** | **0.2** | **1.3** |  |
|  |  |  |  |  |  |
| Dog food | USA | Study mean | -18.2 | 5.1 | Cove et al., 2018 |
| Dry dog food | Brazil | Study mean | -17.1 | 2.9 | de Aro Galera et al., 2019 |
| Wet dog food | Brazil | Study mean | -21.2 | 2.6 | de Aro Galera et al., 2019 |
|  |  | **Dog food study mean** | **-18.8** | **3.5** |  |
|  |  | **Dog food study SD** | **2.1** | **1.4** |  |
|  |  |  |  |  |  |
| Whole milk | USDA estimate | Study mean | -18.7 | 5 | Bostic, 2015 |
| Buns | USDA estimate | Study mean | -25.1 | 2.9 | Bostic, 2015 |
| Beet sugar cookie | USDA estimate | Study mean | -24.6 | 3.3 | Bostic, 2015 |
| Cane sugar cookie | USDA estimate | Study mean | -19 | 3 | Bostic, 2015 |
| Basic biscuit | USDA estimate | Study mean | -25.6 | 3.8 | Bostic, 2015 |
| Muffin | USDA estimate | Study mean | -24.4 | 4.2 | Bostic, 2015 |
| Buttermilk biscuit | USDA estimate | Study mean | -24.7 | 3.8 | Bostic, 2015 |
| Milk chocolate cake | USDA estimate | Study mean | -19.7 | 4.6 | Bostic, 2015 |
| Devils food cake | USDA estimate | Study mean | -19.7 | 4.6 | Bostic, 2015 |
| Dark chocolate cake | USDA estimate | Study mean | -19.7 | 4.5 | Bostic, 2015 |
| Yellow cake | USDA estimate | Study mean | -19.6 | 4.4 | Bostic, 2015 |
| Jelly roll cake | USDA estimate | Study mean | -16.3 | 3.8 | Bostic, 2015 |
| Strawberry jam | USDA estimate | Study mean | -13.2 |  | Bostic, 2015 |
| Prune-raisin brownie | USDA estimate | Study mean | -21.5 | 4.2 | Bostic, 2015 |
| Fudge | USDA estimate | Study mean | -15.6 |  | Bostic, 2015 |
| Salmon, raw-wild | USDA estimate | Study mean | -21.7 | 10.7 | Bostic, 2015 |
| Tuna, canned | USDA estimate | Study mean | -17.5 | 13 | Bostic, 2015 |
| Cod, raw | USDA estimate | Study mean | -18.2 | 14.5 | Bostic, 2015 |
| Catfish, raw | USDA estimate | Study mean | -22.7 | 6.7 | Bostic, 2015 |
| Flatfish, raw | USDA estimate | Study mean | -18.2 | 15.2 | Bostic, 2015 |
| Shrimp, raw | USDA estimate | Study mean | -20.1 | 7.8 | Bostic, 2015 |
| Crab, prepared | USDA estimate | Study mean | -15.9 | 8.7 | Bostic, 2015 |
| Clams, canned | USDA estimate | Study mean | -17.1 | 8.4 | Bostic, 2015 |
| Plant, C3 | Brazil/USA | Study mean | -26.1 | 2.9 | Nardoto et al., 2006 |
| Plant, C4 | Brazil/USA | Study mean | -11.2 | 1.0 | Nardoto et al., 2006 |
| Animal and products | Brazil/USA | Study mean | -16.8 | 4.5 | Nardoto et al., 2006 |
| Seafood | Brazil/USA | Study mean | -19.2 | 12.1 | Nardoto et al., 2006 |
| Fast-food beef | USA | Study mean | -18.0 | 6.5 | Jahren & Kraft, 2008 |
| Fast-food chicken | USA | Study mean | -17.5 | 2.3 | Jahren & Kraft, 2008 |
| Beef patty | USA | Study mean | -15.7 | 6.4 | Chesson et al., 2008 |
|  |  | **Human food study mean** | **-19.4** | **6.2** |  |
|  |  | **Human food study SD** | **3.7** | **3.8** |  |
|  |  |  |  |  |  |
| Anthropogenic input |  | **Corn feed, dog food, and human food mean** | **-16.7** | **4.3** |  |
|  |  | **Corn feed, dog food, and human food SD** | **4.3** | **1.7** |  |

Literature cited

Bostic JN. 2015. Stable isotope variability in the American food supply: implications for dietary reconstruction applications. PhD dissertation, Virginia Polytechnic Institute and State University, Blacksburg, VA.

Boggie MA, Carleton SA, Collins DP, Vradenburg J, Sroka CJ. 2018. Using stable isotopes to estimate reliance on agricultural food subsidies and migration timing for a migratory bird. *Ecosphere* 9: e02083. <https://doi.org/10.1002/ecs2.2083>

Brittain R. 2009. Trophic status, habitat use and climate change impacts on avian species of coastal Georgia. PhD dissertation, School of Public and Environmental Affairs, Indiana University, Bloomington, IN.

Carter WA, Pearson SF, Smith AD, McWilliams SR, Levey DJ. 2021. Seasonal and interspecific variation in frugivory by a mixed resident-migrant overwintering songbird community. *Diversity* 13: 314. <https://doi.org/10.3390/d13070314>

Castelli PM, Reed LM. 2017. Use of stable isotopes to distinguish wild from pen‐raised northern bobwhite. *Wildlife Society Bulletin* 41: 140–145. <https://doi.org/10.1002/wsb.746>

Chesson LA, Podlesak DW, Thompson AH, Cerling TE, Ehleringer JR. 2008. Variation of hydrogen, carbon, nitrogen, and oxygen stable isotope ratios in an American diet: fast food meals. *Journal of Agricultural and Food Chemistry* 56: 4084–4091. <https://doi.org/10.1021/jf0733618>

Cormie AB, Schwarcz HP. 1994. Stable isotopes of nitrogen and carbon of North American white-tailed deer and implications for paleodietary and other food web studies. *Palaeogeography, Palaeoclimatology, Palaeoecology* 107: 227–241. <https://doi.org/10.1016/0031-0182(94)90096-5>

Cove MV, Gardner B, Simons TR, Kays R, O’Connell AF. 2018. Free-ranging domestic cats (*Felis catus*) on public lands: estimating density, activity, and diet in the Florida Keys. *Biological Invasions* 20: 333–344. <https://doi.org/10.1007/s10530-017-1534-x>

Darr RL, Hewitt DG. 2008. Stable isotope trophic shifts in white‐tailed deer. *Journal of Wildlife Management* 72: 1525–1531. <https://doi.org/10.2193/2006-293>

de Aro Galera L, Abdalla Filho AL, Reis LS, de Souza JL, Hernandez YA, Martinelli LA. 2019. Carbon and nitrogen isotopic composition of commercial dog food in Brazil. *PeerJ* 7: e5828. <https://doi.org/10.7717/peerj.5828>

Hayes AA. 2021. Analyses of coyote (*Canis latrans*) consumption of anthropogenic material and dietary composition in urban and non-urban habitats. Thesis, Wright State University, Dayton, OH.

Hutchinson DL, Norr L, Schober T, Marquardt WH, Walker KJ, Newsom LA, Scarry CM. 2016. The Calusa and prehistoric subsistence in central and south Gulf Coast Florida. *Journal of Anthropological Archaeology* 41: 55–73. <https://doi.org/10.1016/j.jaa.2015.10.004>

Jahren AH, Kraft RA. 2008. Carbon and nitrogen stable isotopes in fast food: signatures of corn and confinement. *Proceedings of the National Academy of Sciences* 105: 17855–17860. <https://doi.org/10.1073/pnas.0809870105>

Nardoto GB, Silva S, Kendall C, Ehleringer JR, Chesson LA, Ferraz ES, Moreira MZ, Ometto JP, Martinelli LA. 2006. Geographical patterns of human diet derived from stable‐isotope analysis of fingernails. *American Journal of Physical Anthropology* 131: 137–146. <https://doi.org/10.1002/ajpa.20409>

Newsome SD, Garbe HM, Wilson EC, Gehrt SD. 2015. Individual variation in anthropogenic resource use in an urban carnivore. *Oecologia* 178: 115–128. <https://doi.org/10.1007/s00442-014-3205-2>

Otieno EN, Frenette L. 2017. Stable isotope evidence shows key farmland structure features driving eastern wild turkey food selection. *Ornithological Science* 16: 121–129. <https://doi.org/10.2326/osj.16.121>

Rush SA, Sash K, Carroll J, Palmer B, Fisk AT. 2014. Feeding ecology of the snake community of the Red Hills region relative to management for Northern Bobwhite: assessing the diet of snakes using stable isotopes. *Copeia* 2014: 288–296. <https://doi.org/10.1643/CE-13-083>

Smith S, Mauldin R, Munoz CM, Hard R, Paul D, Skrzypek G, Villanueva P, Kemp L. 2014. Exploring the use of stable carbon isotope ratios in short-lived leporids for local paleoecological reconstruction. *Open Journal of Archaeometry* 2: 5306. <https://doi.org/10.4081/arc.2014.5306>
