# Supplementary figures and images for: Land cover and space use influence coyote carnivory: evidence from stable-isotope analysis
Source: PeerJ. 2024 Jun 4;12:e17457. doi: 10.7717/peerj.17457 (PMC11160434; doi:10.7717/peerj.17457)

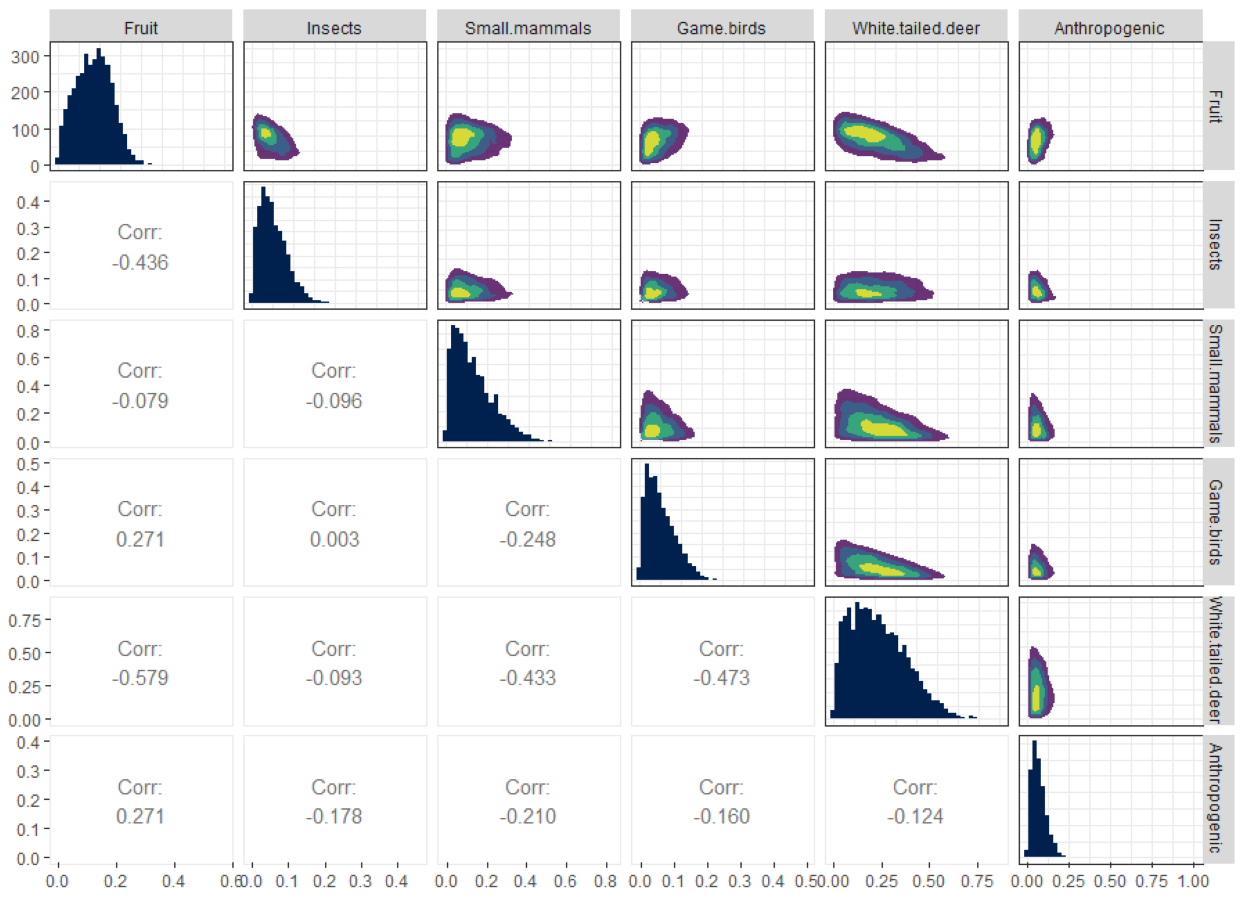

Supplement: Supplemental Information 3 — Histograms are presented along the diagonal showing the estimated proportion of each food source (fruit, insects, small mammals, game birds, white-tailed deer, and anthropogenic) consumed by the individual. The lower diagonal reports the correlation between sources. The contour plots report the relationship among food sources. [file peerj-12-17457-s003.png]

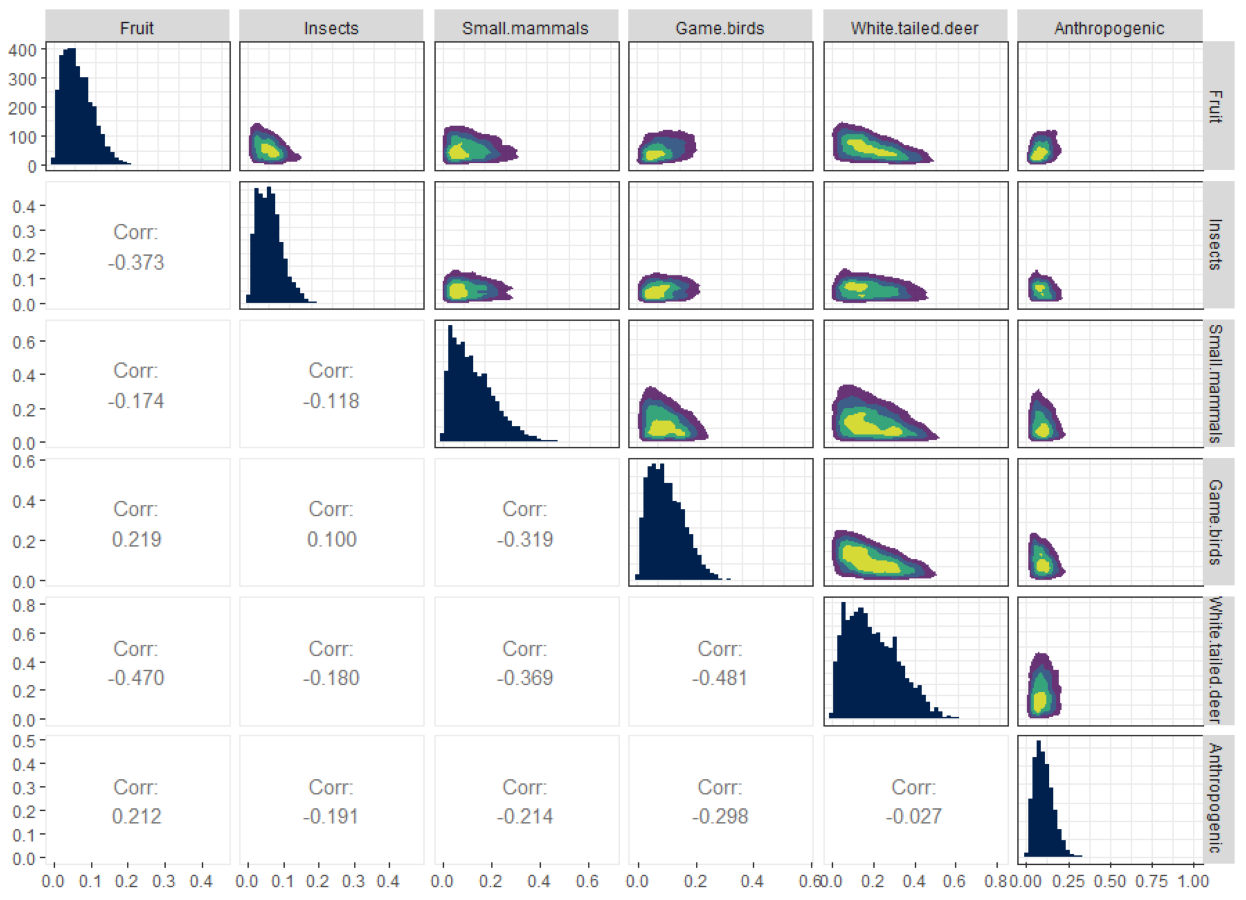

Supplement: Supplemental Information 4 — Histograms are presented along the diagonal showing the estimated proportion of each food source (fruit, insects, small mammals, game birds, white-tailed deer, and anthropogenic) consumed by the individual. The lower diagonal reports the correlation between sources. The contour plots report the relationship among food sources. [file peerj-12-17457-s004.png]

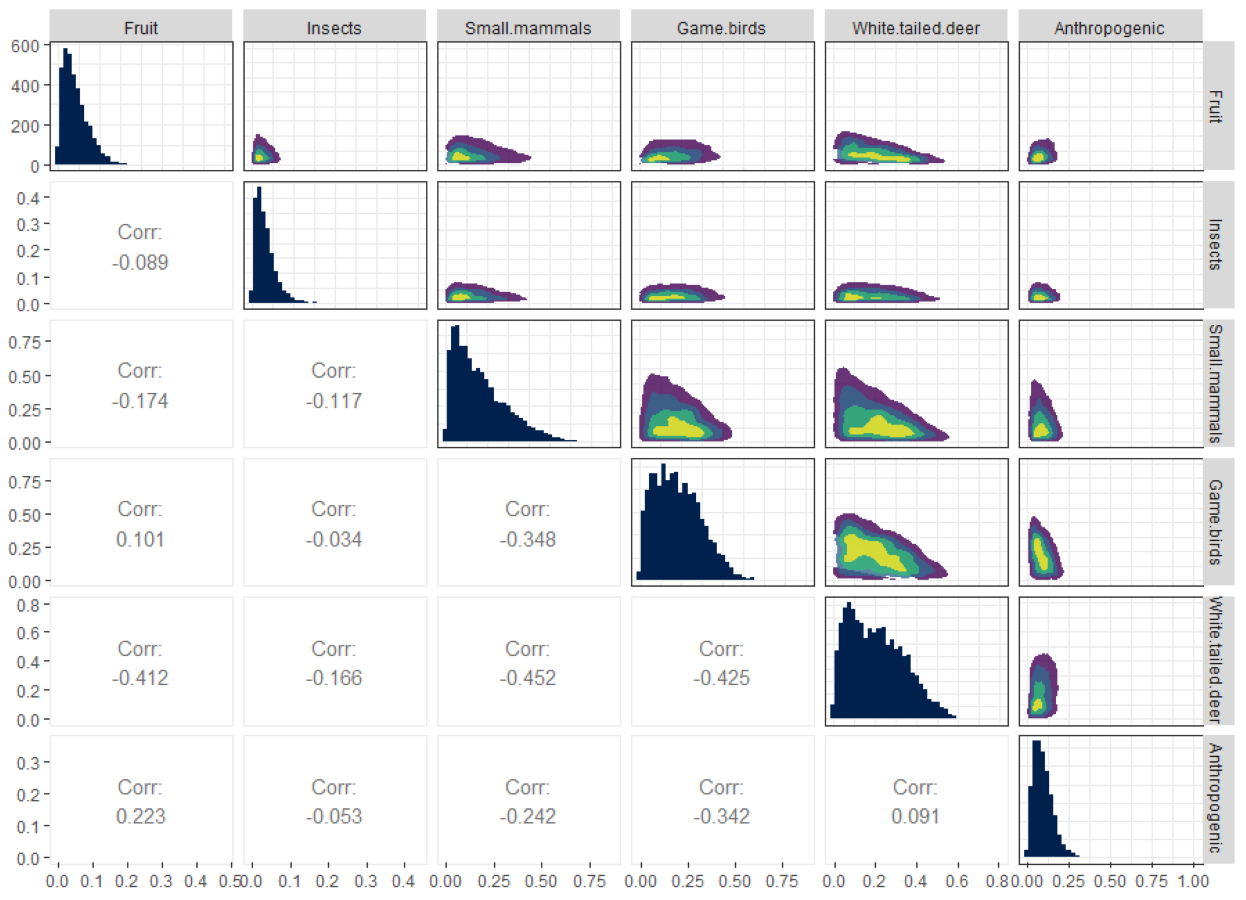

Supplement: Supplemental Information 5 — Histograms are presented along the diagonal showing the estimated proportion of each food source (fruit, insects, small mammals, game birds, white-tailed deer, and anthropogenic) consumed by the individual. The lower diagonal reports the correlation between sources. The contour plots report the relationship among food sources. [file peerj-12-17457-s005.png]

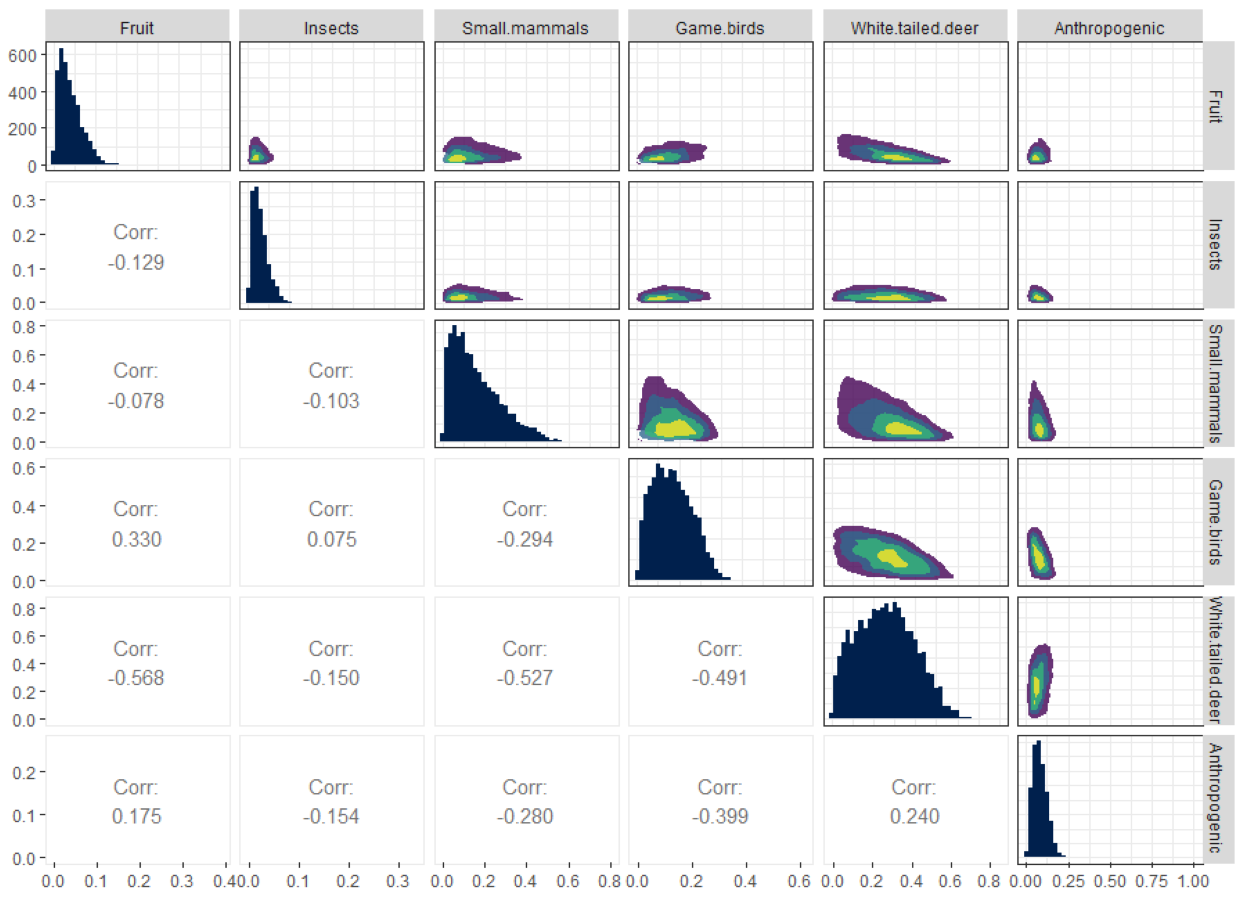

Supplement: Supplemental Information 6 — Histograms are presented along the diagonal showing the estimated proportion of each food source (fruit, insects, small mammals, game birds, white-tailed deer, and anthropogenic) consumed by the individual. The lower diagonal reports the correlation between sources. The contour plots report the relationship among food sources. [file peerj-12-17457-s006.png]

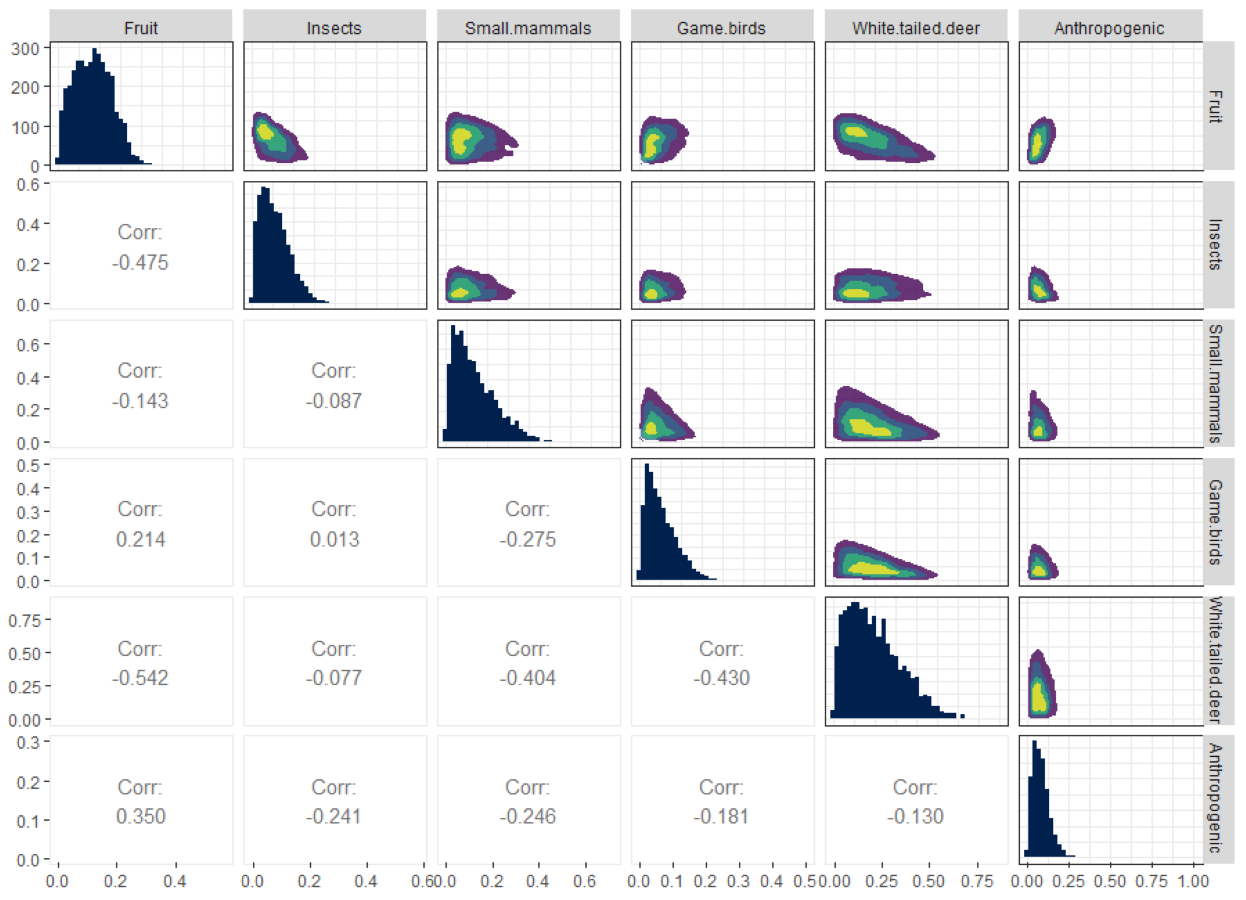

Supplement: Supplemental Information 7 — Matrix plot of food sources consumed by transient coyotes in the Savannah River Area of Georgia and South Carolina, USA, 2016–2017. Histograms are presented along the diagonal showing the estimated proportion of each food source (fruit, insects, small mammals, game birds, white-tailed deer, and anthropogenic) consumed by the individual. The lower diagonal reports the correlation between sources. The contour plots report the relationship among food sources. [file peerj-12-17457-s007.png]
